# Supplementing the early diet of broilers with soy protein concentrate can improve intestinal development and enhance short-chain fatty acid-producing microbes and short-chain fatty acids, especially butyric acid

**DOI:** 10.1186/s40104-022-00749-5

**Published:** 2022-09-08

**Authors:** Qianyun Zhang, Shan Zhang, Shu Wu, Marianne Hjøllund Madsen, Shourong Shi

**Affiliations:** 1 Poultry Institute, Chinese Academy of Agriculture Science, Yangzhou, 225125 China; 2 TripleA a/s, Bjoernkaervej 16, DK-8783 Hornsyld, Denmark

**Keywords:** Broiler, Caecal microbiota, Intestinal development, Short-chain fatty acids, Soy protein concentrate

## Abstract

**Background:**

Research on nutrition in early-life commonly focuses on the maturation of the intestine because the intestinal system is crucial for ensuring continued growth. To explore the importance of early nutrition regulation in animals, soy protein concentrate (SPC) was added to the early diet of broilers to investigate its effects on amino acid digestibility, intestinal development, especially intestinal microorganisms, and broiler metabolites. A total of 192 one-day-old Arbor Acres (AA) male broilers were randomly assigned to two experimental treatments with 8 replicates of 12 birds. The control group was fed a basal diet (control), and the treatment group was fed a basal diet supplemented with 12% SPC (SPC12) during the first 10 d (starter phase). From d 11 to 21 (grower phase) and d 22 to 42 (finisher phase), a basal diet was fed to both treatment groups.

**Results:**

SPC reduced the pH value and acid-binding capacity of the starter diet (*P* < 0.05, d 10); SPC in the early diet enhanced the gizzard weight (*P* < 0.05, d 10 and d 42) and the ileum weight (*P* < 0.05, d 10) and decreased the weight and length of the jejunum (*P* < 0.05, d 10) and the relative length of the duodenum and jejunum (*P* < 0.05, d 10). At the same time, SPC enhanced villus height (*P* < 0.05, d 10) and muscle thickness in the jejunum and ileum (*P* < 0.05, d 10) and increased the number of goblet cells in the duodenum (*P* < 0.05, d 10). Meanwhile, SPC increased the Chao1 index and the ACE index (*P* < 0.05, d 10) and altered the composition of caecal microflora at d 10. SPC also increased the relative abundance of *Alistipes*, *Anaerotruncus*, *Erysipelatoclostridium*, *Intestinimonas* and *Flavonifractor* bacteria (*P* < 0.05, d 10). At the same time, the concentrations of caecal butyric acid and total short-chain fatty acids (SCFAs) were also increased in the SPC12 group (*P* < 0.05, d 10).

**Conclusions:**

In summary, the results showed that supplementing the starter diet of broilers with SPC has a significant effect on the early development of the intestine and the microflora.

**Graphical abstract:**

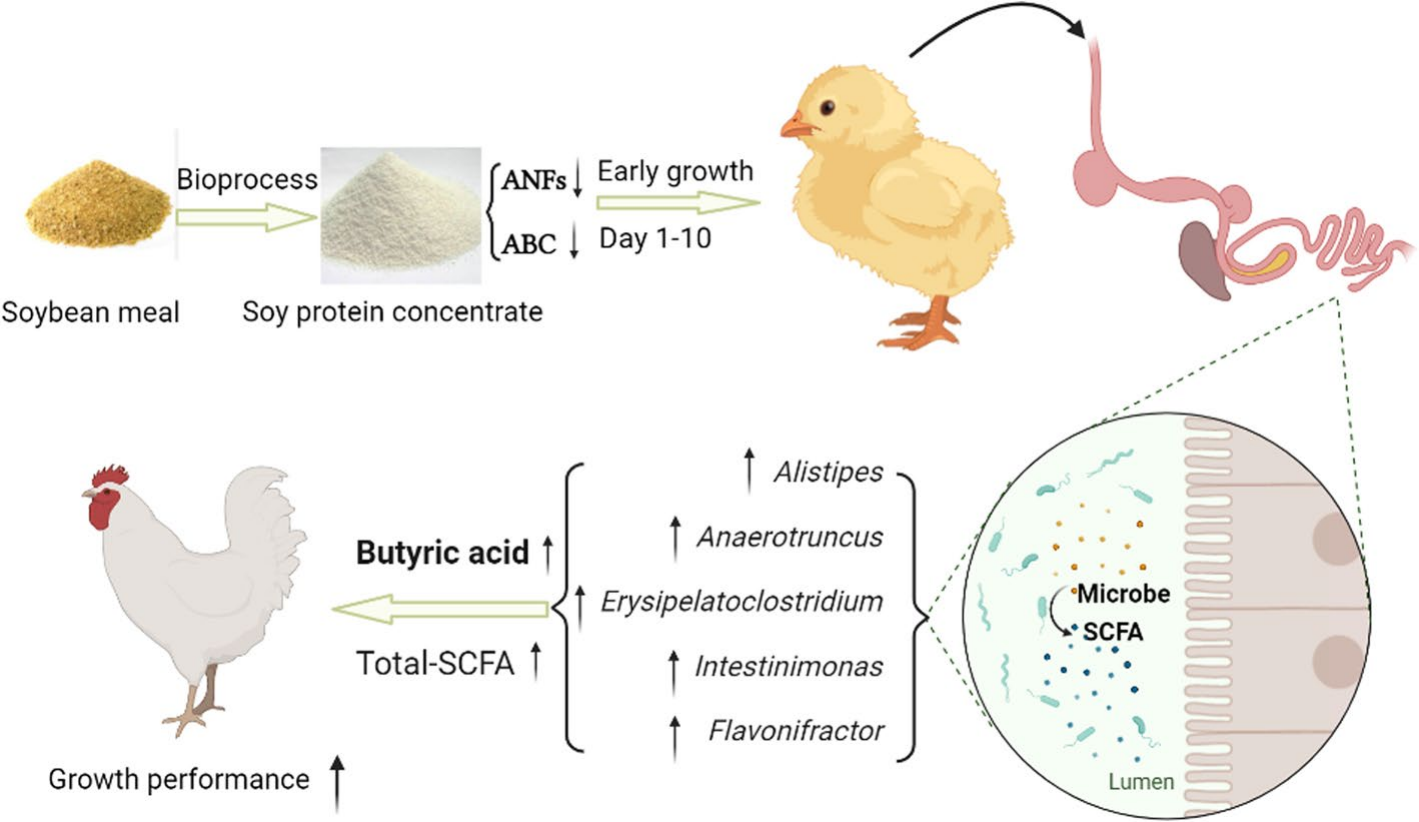

**Supplementary Information:**

The online version contains supplementary material available at 10.1186/s40104-022-00749-5.

## Background

Previous studies have proven that microorganisms in the human intestine may have a fundamental impact on the development of infants and the maturation of the immune system [1, 2]. At the same time, early life intestinal microflora regulates risk factors related to specific adult health status [3], and play a leading role in future health [4]. Therefore, it is imperative to explore the role of microflora in early life [5].

Early life growth has a great impact on an entire organism’s life. Nutritional regulation is an important strategy to improve early growth [6]. Gut microbiota composition is sensitive to early life and is also regulated by diet [7, 8]. Therefore, nutritional strategies to modulate the intestinal microbial ecosystem appear to be a beneficial method to improve intestinal health. Similar to infants, the early life of broilers also plays a decisive role in their later growth. Broilers are slaughtered on average on d 42 in China, and therefore, the first 10 d correspond to a large part of their life (23.81% of the total rearing period). However, it is worth mentioning that the digestive tract of newly hatched chickens is not yet fully developed [9], and the intestinal microbial community is characterized by low diversity and high instability, that is easily affected by external factors such as the intestinal environment [10, 11]. Therefore, the initial developmental stage after hatching is a key phase, which leaves a very narrow window for permanent remodelling of the microflora [12]. Taking into consideration the global prohibition of antibiotics as growth promoting agents, it is a matter of urgency to find other nutritional strategies to regulate intestinal microflora to improve the growth performance of chickens [13].

Soy protein concentrate (SPC) is a type of high-quality soybean protein product obtained by processing soybean meal and removing most of the anti-nutritional factors (ANFs) [14]. Intestinal microflora are affected not only by dietary protein levels [15] but also by dietary protein sources [16]. Unfortunately, most studies have focused on the effects of dietary protein levels on intestinal microorganisms, and only a few researchers have reported the effects of dietary protein sources on intestinal microorganisms [17, 18]. Previous results showed that supplementing the starter diet with SPC can improve the growth performance of broilers [19]. At the same time, previous studies have also pointed out that supplementation with SPC in the early diet can improve the growth performance of broilers by reducing intestinal pathogens and enhancing intestinal health [20]. However, the principle behind how to regulate the intestinal microorganisms of broilers with SPC remains unclear. In this experiment, SPC was added at the early growth phase of broilers (d 1 to 10), and the factors with the potential for improving the growth performance of broilers were analysed: apparent digestibility of amino acids; intestinal development; and caecal microbiota and their metabolites. We hoped that this experiment would provide new knowledge about how to regulate the early nutrition of broilers and provide technical support for the application of SPC in broiler production.

## Methods

### Experimental design

A total of 192 one-day-old chicks – Arbor Acres (AA) male broilers (initial body weight 46.05 ± 0.37 g) – were randomly assigned to two experimental treatments. The control group was fed a basal diet (Control), and the treatment group was fed a basal diet supplemented with 12% SPC (SPC12) during the first 10 d (starter phase). From d 11 to 21 (grower phase) and from d 22 to 42 (finisher phase), a basic diet was fed to all the birds included in the experiment. All diets were formulated to meet or exceed the animal’s nutrient requirements and were isotonic to protein and nutritional levels (NRC 1994) [21]. Ingredient composition and nutrient composition for the experimental diets are provided in Table 1. The SPC added to the diets, AX3 Digest from TripleA, Hornsyld, Denmark, was produced using a new water-based extraction method [22]. The levels of amino acids and proteins in the diets are shown in Additional file 1: Table S1. During the first 10 d, TiO_2_ was added to the experimental diets at a dose of 5 g/kg as an indigestible marker. In addition, we analysed the contents of the main ANFs in the diets, and the results are shown in Table 2.

**Table 1:** Composition and nutrient levels of the diets (air dry basis)

Raw materials	Phases			
	D 1 to 10		D 11 to 21	D 22 to 42
	Control	SPC12		
Ingredient, %				
Corn	50.55	59.20	53.33	57.73
SBM (43%)	42.10	23.40	37.80	33.20
Soy oil	3.40	1.30	4.70	5.50
CaHPO_4_·2H_2_O	1.64	1.86	1.73	1.2
CaCO_3_	1.33	1.30	1.32	1.39
DL-Met	0.24	0.22	0.26	0.24
L-Lys, 98%	0.12	0.10	0.24	0.12
NaCl	0.30	0.30	0.30	0.30
Premix vitamin^a^	0.03	0.03	0.03	0.03
Premix mineral^b^	0.20	0.20	0.20	0.20
Choline chloride (70%)	0.09	0.09	0.09	0.09
SPC	0.00	12.00	0.00	0.00
Total	100	100	100	100
Nutrient levels^c^				
ME, kcal/kg	2950	2950	3050	3150
CP, %	22.50	22.50	21.00	19.00
Ca, %	1.00	1.00	1.00	0.90
K, %	1.18	0.78	1.12	1.01
Na, %	0.13	0.16	0.13	0.14
Total P, %	0.69	0.70	0.69	0.58
NPP, %	0.45	0.45	0.45	0.35
D-Lys, %	1.18	1.19	1.19	1.00
D-Met, %	0.56	0.55	0.55	0.50

^a^Premix vitamin provided per kilogram of diet: vitamin A (retinyl palmitate), 8000 IU; vitamin D_3_ (cholecalciferol), 1000 ICU; vitamin E (D/L-α-tocopheryl acetate), 20 IU; vitamin K_3_ (menadione sodium bisulfate complex), 0.50 mg; vitamin B_1_, 2.00 mg; vitamin B_2_, 8.00 mg; vitamin B_6_, 3.50 mg; vitamin B_12_ (cobalamin), 10.00 μg; niacin, 35.00 mg; calcium pantothenic, 10.00 mg; folic acid, 0.55 mg; and biotin, 0.18 mg

^b^Premix mineral provided per kilogram of diet: Fe 80.00 mg; Mn 100.00 mg; Zn 80.00 mg; I 0.70 mg; Se 0.30 mg; and Cu 8.00 mg

^c^The nutrient level is the calculated value, where K and Na are measured

**Table 2:** Analysis of the main antinutritional factors in the diet

Items	Phases			
	D 1 to 10		D 11 to 22	D 22 to 42
	Control	SPC12		
Glycinin, mg/g	10.0	4.5	14.0	14.0
β-Conglycinin, mg/g	3.0	2.2	4.0	3.1
Lectin, mg/kg	15	10	12	10
Raffinose, g/100 g	0.55	0.34	0.37	0.35
Stachyose, g/100 g	1.31	0.74	1.47	1.49
Trypsin inhibitor, TIU/g	2150	1402	2450	2300

### Bird management and sample collection

Feeding management during the trial is addressed in a different study [19], and all feeding management was carried out according to the feeding management standards for broilers.

On d 10, 12 broilers in each pen were anaesthetized by intravenous injection of chloral hydrate. The contents of the terminal ileum were collected starting at 15 cm and onwards in a centrifuge tube and quickly frozen in liquid nitrogen. The samples were kept at − 80 °C until the determination of apparent digestibility of the amino acids.

On d 10 and 42, one broiler was selected from each replicate. After decapitation and bleeding, the abdominal cavity was opened gently, and the whole digestive tract was removed. The contents of the crop, gizzard, glandular stomach, pancreas, duodenum, jejunum, ileum and caecum were extracted. The pancreas was placed into a 5-mL centrifuge tube and frozen in liquid nitrogen for subsequent determination of enzyme activity. Subsequently, 1 cm of duodenum, jejunum and ileum was taken and placed into a tube filled with 10% formaldehyde solution for the analysis of intestinal mucosal morphology. Afterwards, digesta from the gizzard, duodenum, jejunum and ileum were pooled into one bag, and the pH was subsequently measured (Table S2). On d 10 and 42, each replicate of 8 birds was weighed before sample collection: the weight of the gizzard and the length and weight of the duodenum, jejunum and ileum were recorded; the relative length and relative weight of each intestinal segment were calculated. Finally, the caecal contents were stored in two cryopreservation tubes for 16S rDNA sequencing and metabonomic analysis.

### Sample processing and chemical analysis

#### pH values and acid-binding capacity of starter diets

The acid-binding capacity (ABC) and pH of the starter diet were determined using the methods reported in a previous study [23]. All pH measurements were made using a laboratory pH metre (PHM 220, Radiometer, Copenhagen), which was calibrated using certified pH = 4.0 and pH = 7.0 buffer solutions. A 100-g sample of feed was suspended in 200 mL of distilled water and continuously stirred with a magnetic stirrer. Titrations were performed by adding acid (0.1 mol/L HCl) in variable increments until pH = 4 or pH = 3. Initial pH and all further readings taken during the titration were recorded after equilibration for 3 min. Three replicates are required for each feed. ABC was calculated as the amount of acid in milliequivalents (meq) required to lower the pH of 1 kg of sample to (a) a pH of 4.0 (ABC-4) and (b) a pH of 3.0 (ABC-3).

#### Apparent total tract digestibility of amino acids

Amino acids were determined using an amino acid analyser (Biochrom, Version 30, Biochrom Ltd., Cambridge, UK). In brief, samples were oxidized in a mixture of hydrogen peroxide and phenolic formic acid solution and then left to react for 16 h at 0 °C for the oxidation of cystine and methionine. Samples were subsequently neutralised with a spatula tip of sodium disulfide and then hydrolysed with 6 mol/L HCl (containing phenol) for 23 h at 110 °C. The amino acids were then detected on an ion-exchange column, and the chromatograms were integrated using the OPENLAB software with amino acids simultaneously detected at 570 nm and 440 nm.

$$\mathrm{AID}\ \left(\%\right)=\left(1-\frac{{\mathrm{TiO}}_2\ \mathrm{in}\ \mathrm{diet}\ \left(\%\right)}{{\mathrm{TiO}}_2\ \mathrm{in}\ \mathrm{digesta}\ \left(\%\right)}\times \frac{\mathrm{Digesta}\ \mathrm{nutrient}\ \left(\%\right)}{\mathrm{Diet}\ \mathrm{nutrient}\ \left(\%\right)}\right)\times 100$$

#### Pancreatic enzyme activity

The activities of trypsin and chymotrypsin were determined using the method of O’Sullivan et al. [24]. Pancreatic homogenate supernatant was activated with an equal volume of 0.1% enterokinase (E0632, Sigma–Aldrich) for 60 min in a 40 °C water bath. Trypsin and chymotrypsin activities were based on 1 mmol N-α-benzoyl-DL-arginine-4-nitroanilide hydrochloride (BAPNA, B4875, Sigma–Aldrich) and 1 mmol N–glutaryl-L-phenylalanine-4-nitroanilide (GPNA, G2505, Sigma–Aldrich) as substrates, respectively. The substrate solution (including 3.7 mmol/L Tris-HCl buffer (pH = 7.8), 6 mmol/L CaCl_2_ and 25 mg/mL dimethyl sulfoxide) was left to react at 40 °C for 60 min, and then the reaction was terminated with 30% acetic acid. The absorbance of p-nitroaniline was determined at 410 nm. The standard curve was created with the absorbance values of p-nitrotoluene at a wavelength of 410 nm at concentrations of 0, 0.05, 0.10, 0.15, 0.20 and 0.25 μmol, and the activities of trypsin and chymotrypsin were extrapolated. One active unit of trypsin and chymotrypsin is defined as the amount of enzyme consumed to produce 0.1 μmol p-nitrotoluene. It is expressed as u/g pancreatic protein. The protein content of the pancreas was determined by Coomassie brilliant blue assay [25].

The pancreatic general proteolytic (GP) activity was determined according to the method of Susbilla et al. [26]. Pancreatic tissue was homogenized in Ringer’s solution (pH = 7.4), and then the supernatant was activated with enterokinase (E0632, Sigma–Aldrich) under frozen conditions for 2 h. Two percent casein (C8654, Sigma–Aldrich) was dissolved in 0.2 mol sodium phosphate buffer at pH 8.0 as a substrate. The substrate was added to the centrifuge tube with supernatant and then incubated in 41 °C oscillating water baths for 20 min, and the reaction was terminated with 5% trichloroacetic acid. The reaction solution was filtered with Whatman 1 filter paper. Na_2_CO_3_ (0.5 mol) was added to the filtrate, and the free tyrosine content was determined by Folin-Ciocalteu’s solution. The tyrosine standard was used to draw the standard curve and calculate the activity of the general pancreatic proteolytic activity.

#### Intestinal mucosal morphology and goblet cells

Fixed duodenum, jejunum and ileum tissues of eight broilers per feeding group were cut into a longitudinal cross section and embedded in paraffin wax. The tissues (5 μm) were then sectioned (Leica, RM2016, Shanghai) and stained with haematoxylin and eosin (H&E) for morphological measurements. Each slide was captured with a Nikon DS-U3 digital camera coupled to a Nikon Eclipse E100. NIS-Elements F software was used for image capturing. Morphometric analysis was performed by Image-Pro Plus 6.0 software (Media Cybernetics, Inc., Rockville, MD, USA). The evaluated morphometric indices were villus height (VH, from the tip of the villus to the crypt), crypt depth (CD, from the base of the villus to the submucosa) and villus height to crypt depth ratio (VCR) [27]. Morphometric measurements were performed on 10 well-oriented and intact villi and 10 crypts chosen from the duodenum, jejunum and ileum [28]. Muscularis thickness was measured at the middle of the villus and crypt. All the indicators correspond to each other. Goblet cells were read at 200 times magnification. The number of goblet cells in 3 villi was read from each section, and the length of the villi epithelium was measured. Finally, the number of goblet cells per unit length was calculated.

#### DNA extraction and sequencing library construction

Approximately 200 mg samples were taken from each tube of the chicken caecal content and homogenized using a three-minute bead beating procedure at 30 Hz. Bacterial genomic DNA was extracted using the CATB method. The quality and integrity of each DNA sample was determined by electrophoresis in a 1% agarose gel with Tris-acetate-EDTA (TAE) buffer. The DNA concentration was quantified using a NanoDrop ND-2000 spectrophotometer (Thermo Scientific, Wilmington, USA). Thirty-two libraries were constructed and sequenced using the Illumina MiSeq sequencing platform. PCR amplifications were conducted from each sample to produce the V3-V4 hypervariable region (341F: 5'-CCTAYGGGRBGCASCAG-3', 806R: 5'-GGACTACNNGGGTATCTAAT-3') of the 16S rDNA gene, according to previously described methods [29, 30]. Sequencing was performed at Novogene Bioinformatics Technology Co., Ltd. (Beijing, China).

#### DNA sequence processing and analysis

After removing barcode and primer sequences, the reads of each sample were spliced using FLASH V 1.2.7 (http://ccb.jhu.edu/software/FLASH/index.shtml) [31]. Uparse software V 7.0.1001 (http://www.drive5.com/uparse/) [32] was used to cluster the high-quality sequences of all samples. By default, the sequences were clustered into operational taxonomic units (OTUs) with 97% identity. At the same time, the most frequent OTU sequence was selected as the representative OTU sequence, and was annotated. After homogenizing the sample data, Qiime software V 1.9.1 (http://qiime.org/) was used to calculate the alpha diversity. R software V 2.15.3 (http://www.r-project.org/) was used to draw principal coordinate analysis (PCoA) diagrams and Spearman correlation analysis. Finally, a nonparametric test was used to analyse the difference in microbiota.

#### Analysis of short-chain fatty acids

An appropriate amount of caecal content was suspended in 2 mL of 25% phosphoric acid solution and homogenized by vortexing for 2 min. Two millilitres of ether was added to the extract for 10 min, which was then centrifuged for 20 min at 4 °C and 13,000×*g*. After centrifugation, the ether phase was collected, and the above operation was repeated once. The two extracts were combined with volatilization and the volume was brought up to 2 mL for injection analysis. The SCFA content was determined using a gas chromatograph mass spectrometer (7890B-7000D, Agilent, USA) equipped with a flame ionization detector. A 25 m × 0.20 mm × 0.40 μm column (HP-INNOWAX, Agilent, USA) was used to separate the SCFAs. The temperature of the injection port was 240 °C, and there was no split. The carrier gas was nitrogen, and the flow rate was 1.0 mL/min. The mass spectrum conditions were as follows: the temperatures of the ion source and transmission line were 200 °C and 250 °C, respectively. The EI ion source was used, and the bombardment voltage was 70 eV. The column temperature program was as follows: the column temperature was held at 100 °C for 5 min, then heated at 5 °C/min to 150 °C, and finally heated at 30 °C/min to 250 °C (held for 30 min).

Acetic acid, propionic acid, isobutyric acid, butyric acid, isovaleric acid and valeric acid standards were weighed, acetonitrile was used to prepare a certain concentration gradient, which was detected by GC–MS, and a standard curve was made. The standard curves of each standard were *y* = 331,648.5727*x*-2.7102782 (*R*^2^ = 0.9999), *y* = 256,759.9852*x*-1536.5145 (*R*^2^ = 0.9991), *y* = 476,823.311*x*-3752.1943 (*R*^2^ = 0.9993), *y* = 1,452,462.3568*x*-3568.0179 (*R*^2^ = 0.9998), *y* = 1,224,498.7106*x*-2860.8646 (*R*^2^ = 0.9998) and *y* = 1,792,785.5396*x*-1980.5223 (*R*^2^ = 0.9996). The content of short-chain fatty acids in each sample was calculated by their respective standard curves.

### Statistical analysis

An individual pen served as the experimental unit. Data were analysed by independent-samples T test using SPSS 21.0 for Windows (SPSS Inc., Chicago, IL, USA), and differences were considered statistically significant at *P* < 0.05. The results are expressed as the mean and SE.

## Results

### Acid binding capacity and pH value

Table 3 shows the effect of SPC supplementation in starter diets on acid binding capacity and pH value. Compared with the control group, the values of pH, ABC-4 and ABC-3 were decreased (*P* < 0.01) in the SPC12 group.

**Table 3:** Effects of SPC in the starter diet on acid binding capacity of broilers at 1-10 d

Items	Group		*P *value
	Control	SPC12	
pH	5.96 ± 0.00	5.23 ± 0.00	< 0.01
ABC-4	138.74 ± 1.27	108.58 ± 1.14	< 0.01
ABC-3	308.98 ± 1.75	273.16 ± 1.14	< 0.01

The values in the table are the means ± SEM (*n* = 3); *Control* Control group, *SPC12* Supplementation with 12% SPC to the starter diet group, *ABC* Acid binding capacity

### Pancreatic enzyme activity and apparent amino acid digestibility

The effect of a starter diet supplemented with SPC on the pancreatic enzyme activity of broilers is shown in Table 4. Compared with the control group, pancreatic enzyme activity was unchanged in the SPC12 group at d 10 or 42 (*P* > 0.05). The effect of a starter diet supplemented with SPC on the apparent amino acid digestibility of broilers is shown in Table 5. Compared with the control group, the apparent digestibility of amino acids was unchanged in the SPC12 group at d 10 (*P* > 0.05).

**Table 4:** Effects of SPC in the starter diet on pancreatic enzyme activity of broilers

Items	Group		*P *value
	Control	SPC12	
D 10			
GP-pancreas, U/g of protein	1442.31 ± 114.07	1398.51 ± 159.59	0.827
Chymotrypsin, U/g of protein	1.99 ± 0.31	2.55 ± 0.20	0.148
Trypsin, U/g of protein	39.72 ± 3.17	43.26 ± 3.17	0.416
D 42			
GP-pancreas, U/g of protein	961.23 ± 51.94	950.27 ± 80.41	0.914
Chymotrypsin, U/g of protein	1.24 ± 0.41	0.96 ± 0.20	0.573
Trypsin, U/g of protein	35.44 ± 2.79	34.15 ± 1.52	0.702

The values in the table are the means ± SEM (*n* = 8); *Control* Control group, *SPC12* Supplementation with 12% SPC to the starter diet group, *GP-pancreas* General proteolytic activity

**Table 5:** Effects of SPC in the starter diet on apparent digestibility of amino acids at the terminal ileum of broilers (dried matter basis), %

Items	Groups		*P *value
	Control	SPC12	
Methionine	77.04 ± 0.95	78.92 ± 1.64	0.457
Cystine	56.20 ± 1.66	59.26 ± 2.26	0.150
Methionine + Cystine	68.94 ± 1.22	70.86 ± 1.89	0.436
Lysine	68.31 ± 1.24	70.04 ± 2.31	0.580
Threonine	55.29 ± 1.40	57.44 ± 2.86	0.519
Arginine	76.97 ± 0.90	78.66 ± 1.53	0.426
Isoleucine	68.41 ± 0.99	68.55 ± 2.10	0.941
Leucine	70.41 ± 0.96	69.58 ± 2.02	0.722
Valine	66.22 ± 1.08	68.07 ± 2.10	0.572
Histidine	70.39 ± 1.02	71.33 ± 1.91	0.712
Phenylalanine	72.73 ± 0.87	74.21 ± 1.65	0.545
Tyrosine	70.93 ± 0.99	71.22 ± 2.21	0.567
Glycine	60.97 ± 1.13	62.22 ± 2.40	0.715
Serine	62.57 ± 1.24	65.95 ± 2.28	0.188
Proline	68.68 ± 1.08	71.50 ± 1.76	0.497
Alanine	65.27 ± 1.18	67.32 ± 2.21	0.434
Aspartate	67.71 ± 0.92	68.45 ± 1.96	0.815
Glutamate	75.56 ± 0.81	77.07 ± 1.50	0.403
Total amino acids contain ammonia	68.98 ± 1.00	70.29 ± 1.96	0.567
Total amino acids	69.24 ± 0.99	70.33 ± 1.96	0.992

The values in the table are the means ± SEM (*n* = 8); *Control* Control group, *SPC12* Supplementation with 12% SPC to the starter diet group

### Intestinal development

**Fig. 1:**
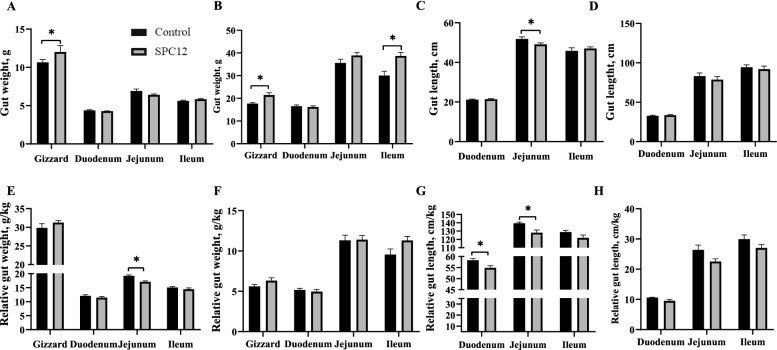
Effects of SPC supplementation in the starter diet on intestinal development of broilers. The values in the histogram are the means ± SEM (*n* = 8), *P < 0.05. Control = control group; SPC12 = supplementation with 12% SPC to the starter diet group; **A** Gut weight at 10 d; **B** Gut weight at 42 d; **C** Gut length at 10 d; **D** Gut length at 42; **E** Relative gut weight at 10 d; **F** Relative gut weight at 42 d; **G** Relative gut length at 10 d; **H** Relative gut length at 42

**Fig. 2:**
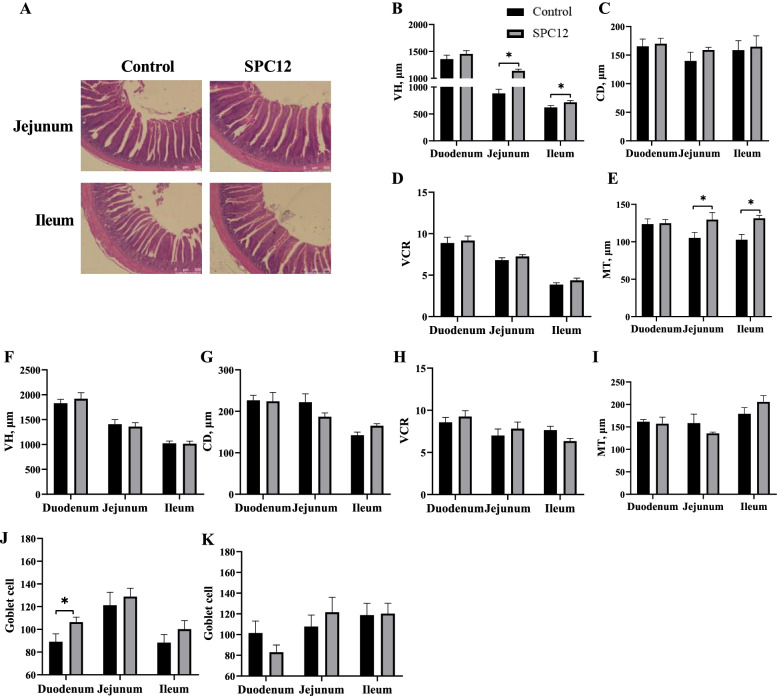
Effects of SPC supplementation in the starter diet on intestinal morphology of broilers. The values in the histogram are the means ± SEM (*n* = 8), *P < 0.05. Control = control group; SPC12 = supplementation with 12% SPC to the starter diet group; **A** Picture of jejunum and ileum at d 10.; **B** Gut villus height at d 10; **C** Gut crypt depth at d 10; **D** Gut VCR at d 10; E = gut muscular thickness at d 10; **F** Gut villus height at d 42; **G** Gut crypt depth at d 42; **H** Gut VCR at d 42; **I** Gut muscular thickness at d 42; **J** Goblet cell at d 10; **K** Goblet cell at d 42; VH = villus height; CD = crypt depth; MT = muscle thickness; VCR = the ratio of villus height to crypt depth

The results regarding the effects of SPC supplementation on intestinal development of broilers are presented in Fig. 1. Compared with the control group, the absolute weight of the gizzard in the SPC12 group was increased at d 10 and 42 (*P* < 0.05), whereas the relative weight and length of the jejunum was decreased in the SPC12 group at d 10 (*P* < 0.05). In addition, the absolute ileum weight was increased in the SPC12 group at d 42 (*P* < 0.05). Figure 2A-E shows the intestinal morphology of broilers fed SPC in the starter phase. At d 10, compared with the control group, the villus height of the jejunum and the muscle thickness of the ileum were increased in the SPC12 group (*P* < 0.05). At d 42, compared with the control group, the VCR of the ileum was decreased in the SPC12 group (*P* < 0.05). Supplementing SPC in the starter diet did not change other intestinal morphology indices (*P* > 0.05). The effects of SPC in a starter diet on the number of goblet cells in the intestinal tract are shown in Fig. 2F-I. Compared with the control group, the number of goblet cells in the duodenum was increased in the SPC12 group at d 10 (*P* = 0.05) (Fig. 2J). The number of goblet cells in the other segments of the intestine was not affected by SPC in the starter diet at d 10 or 42 (*P* > 0.05) (Fig. 2K).

### Intestinal microbial diversity and community

**Fig. 3:**
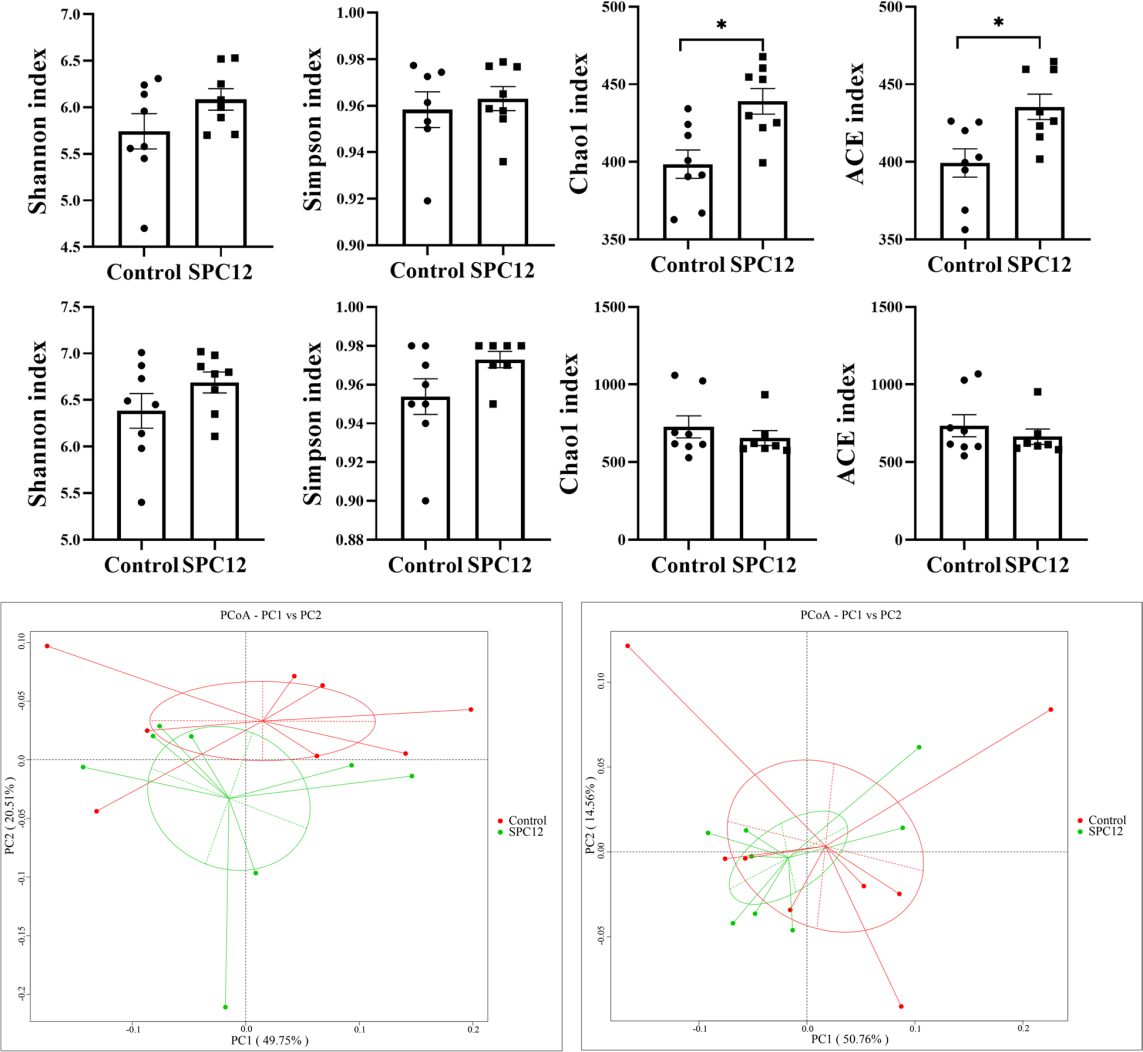
Effects of SPC supplementation in the starter diet on the diversity of caecal microorganisms of broilers. The values in the histogram are individual sample values from maximum to minimum (*n* = 8), *P < 0.05. Control = control group; SPC12 = supplementation with 12% SPC to the starter diet group; **A** Shannon index at d 10; **B** Simpson index at d 10; **C** Chao1 index at d 10; **D** ACE index at d 10; **E** Shannon index at d 42; **F** Simpson index at d 42; **G** Chao1 index at d 42; **H** ACE index at d 42; **I** PCoA at d 10; **J** PCoA at d 42

**Fig. 4:**
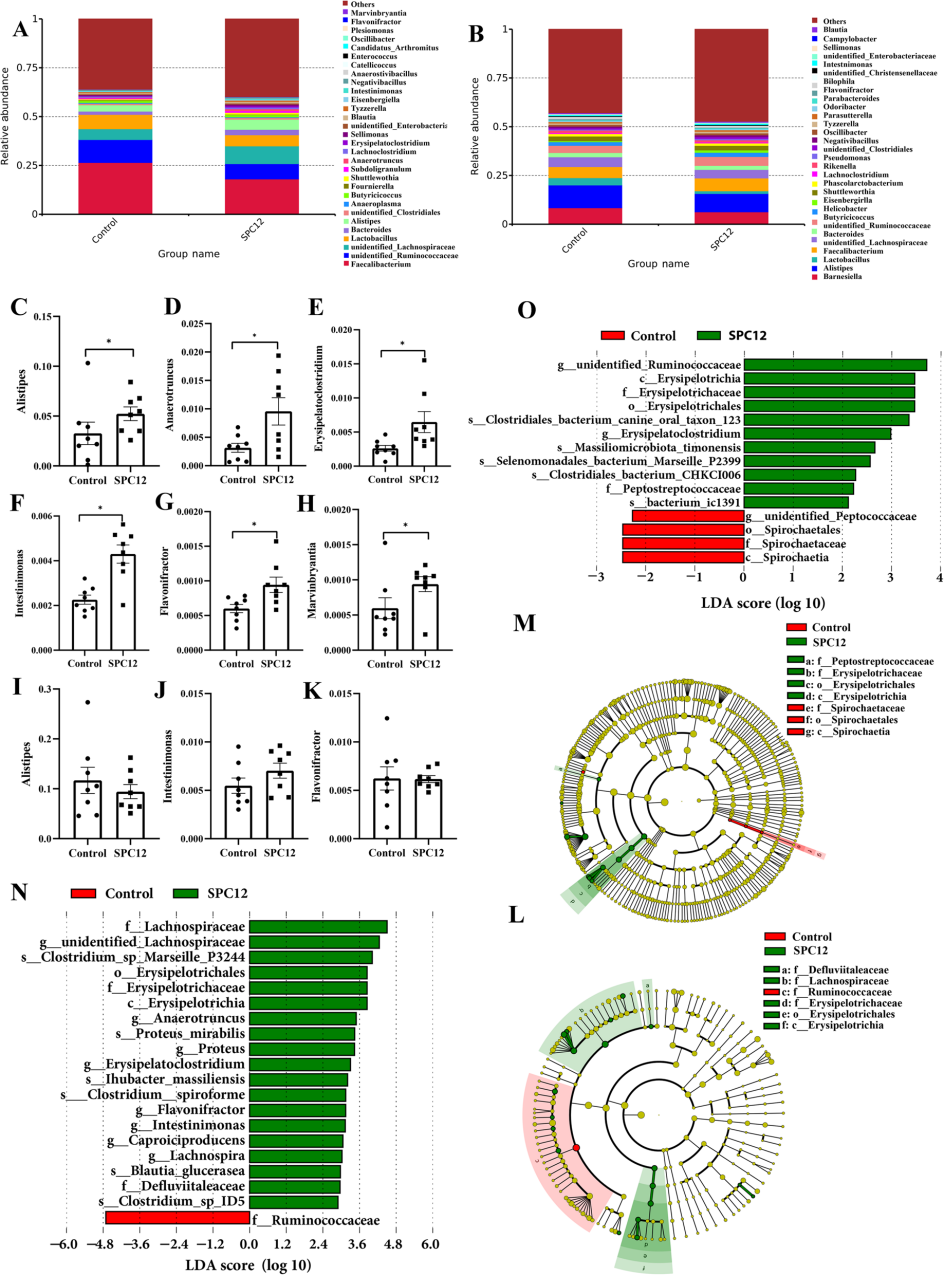
Effects of SPC supplementation in the starter diet on the relative abundance of the top 30 bacteria at the genus level of broilers. The values in the histogram are individual sample values from maximum to minimum (*n* = 8), *P < 0.05. Control = control group; SPC12 = supplementation with 12% SPC to the starter diet group. **A** Relative abundance of caecal microflora in the top 30 at d 10; **B** Relative abundance of caecal microflora in the top 30 at d 42; **C** Relative abundance of *Alistipes* at d 10; **D** Relative abundance of *Anaerotruncus* at d 10; **E** Relative abundance of *Erysipelatoclostridium* at d 10; **F** Relative abundance of *Intestinimonas* at d 10; **G** Relative abundance of *Flavonifractor* at d 10; **H** Relative abundance of *Marvinbryantia* at d 10; **I** Relative abundance of *Alistipes* at d 42; **J** Relative abundance of *Intestinimonas* at d 42. **K** Relative abundance of *Flavonifractor* at d 42; **L** and **N** LEfSe analysis of caecal microflora at d 10; **M** and **O** LefSe analysis of caecal microflora at d 42

Alpha-diversity analysis showed that SPC12 increased the Chao1 index and ACE index of broilers at d 10 (*P* < 0.05). However, there was no significant effect on broilers at d 42 (*P* > 0.05), suggesting that the overall bacterial richness of caecal microbiota was increased by SPC (Fig. 3A-H). Supplementing 12% SPC in the starter diet changed the microbial composition of broilers, but it had no significant effect on the composition of intestinal microorganisms at d 42 (Fig. 3I and J). The relative abundance of microorganisms in the TOP 30 was analysed. At d 10, the relative abundance of *Faecalibacterium* and unidentified*-Ruminococcaceae*, unidentified*-Lachnospiraceae*, *Lactobacillus*, *Bacteroides*, *Alistipes* and *Butyricicoccus* was greater than 1% (Fig. 4A). We further analysed the differential bacteria of TOP 30 with a *T* test and found that the relative abundance of *Alistipes*, *Anaerotruncus*, *Erysipelatoclostridium*, *Intestinimonas*, *Flavonifractor* and *Marvinbryantia* increased in the SPC12 group (Fig. 4C-H and Table S3). At d 42, the relative abundance of *Barnesiella*, *Alistipes*, *Lactobacillus*, *Faecalibacterium*, unidentified*-Lachnospiraceae*, *Bacteroides*, unidentified*-Ruminococcaceae* and *Butyricicoccus* was more than 1% (Fig. 4B). At the same time, there was no significant difference observed in the relative abundance between *Alistipes*, *Intellinimonas* and *Flavonifractor*; in addition, *Anaerotruncus*, *Erysipelatoclostridium* and *Marvinbryantia* were not in the Top 30 at d 42 (Fig. 4I-K and Table S4).

The significant and differentially abundant OTUs for the entire microbiota at the phylum to genus levels were analysed by linear discriminant analysis effect size (LEfSe) (LDA > 2.0; Fig. 4(L and N, and M and O)), and the results indicated that there were dramatic differences in microbial composition between the SPC12 groups and the control group. At d 10, at the family level, the abundance of Lachnospiraceae, Erysipelotrichaceae and Defluvialeaceae was higher in the SPC12 group, while the abundance of Ruminococcaceae was higher in the control group. At the genus level, the abundance of unidentified*-Lachnospiraceae*, *Anaerotruncus*, *Proteus*, *Erysipelatoclostridium*, *Flavonifractor*, *Intestinimonas*, *Caproiciproducens* and *Lachnospira* was higher in the SPC12 group. At d 42, at the family level, the abundance of Erysipelotrichaceae and Peptostreptococcaceae was higher in the SPC12 group, while the abundance of Spirochaetaceae was higher in the control group. At the genus level, the abundance of unidentified*-Ruminococcaceae* and *Erysipelatoclostridium* was higher in the SPC12 group, while the abundance of unidentified*-Peptococcaceae* was higher in the control group.

### Short-chain fatty acids 

**Fig. 5:**
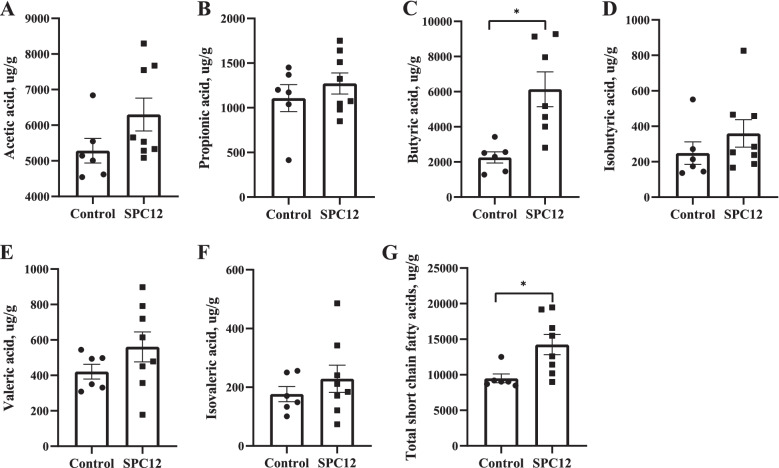
Effects of SPC supplementation in the starter diet on short-chain fatty acids of broilers at d 10. The values in the histogram are individual sample values from maximum to minimum (*n* = 8), *P < 0.05. Control = control group; SPC12 = supplementation with 12% SPC to the starter diet group. **A** acetic acid; **B** propionic acid; **C** butyric acid; **D** Isobutyric acid; **E** Valeric acid; **F** Isovaleric acid; **G** Total short-chain fatty acids

Short-chain fatty acids in the caecum were determined at d 10 (Fig. 5). The results showed that supplementation with 12% SPC in the starter diet increased the content of butyric acid and total SCFAs (*P* < 0.05), but there was no significant effect on the content of other SCFAs in the caecum.

### Correlation analysis

**Fig. 6:**
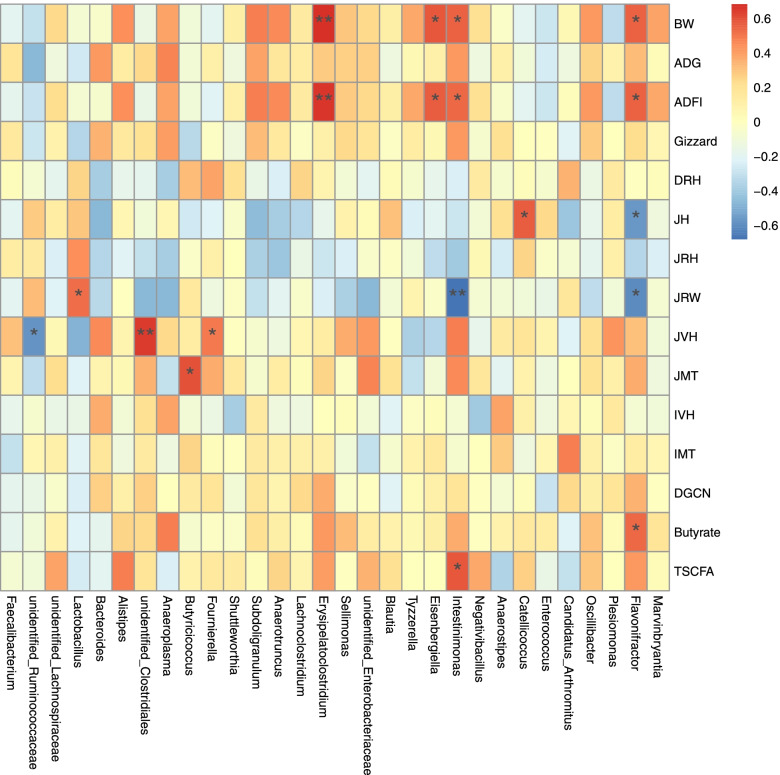
Effects of SPC supplementation in the starter diet on correlation analysis between difference indices and microorganisms in the top 30 genera of broilers at d 10. *P < 0.05; **P < 0.01 (following Spearman correlation analysis). BW = body weight; ADG = average daily gain; ADFI = average daily feed intake; DRH = duodenum relative height; JH = jejunum height; JRH = jejunum relative height; JRW = jejunum relative weight; JMT = jejunum muscular thickness; IVH = ileum villus height; IMT = ileum muscular thickness; DGCN = duodenum goblet cell number; TSCFA = total short-chain fatty acids

Spearman correlation analysis was conducted to estimate the association between the phylotypes in the gut microbiota and different phenotypes of broilers (Fig. 6). As shown in the heatmap, the abundance of unidentified*-Ruminococcaceae* exhibited negative relationships with JVH (*P* < 0.05), the abundance of *Intestinimonas* exhibited negative relationships with JRW (*P* < 0.01), and the abundance of *Flavonifractor* exhibited negative relationships with JH and JRW (*P* < 0.05). Conversely, the abundance of unidentified*-Clostridiales* (*P* < 0.01) and *Fournierella* (*P* < 0.05) correlated positively with JVH, the abundance of *Lactobacillus* correlated positively with JRW (*P* < 0.05), the abundance of *Butyricicoccus* correlated positively with JMT (*P* < 0.05), the abundance of *Erysipelatoclostridium* correlated positively with BW and ADFI (*P* < 0.01), the abundance of *Catellicoccus* correlated positively with JH (*P* < 0.05), and the abundance of *Eisenbergiella*, *Intestinimonas* and *Flavonifractor* correlated positively with BW and ADFI (*P* < 0.05). In addition, the abundance of *Intestinimonas* correlated positively with T-SCFA (*P* < 0.05), and the abundance of *Flavonifractor* correlated positively with butyrate (*P* < 0.05),

## Discussion

Previous studies have shown that supplementing starter diets with 12% SPC improved the growth performance of broilers [19]. Therefore, this study continued the previous work and studied the digestive tract enzyme activity, amino acid digestibility, intestinal development, and intestinal microorganisms of the SPC12 group.

### Adding SPC to the broiler starter diet reduced the acid-binding capacity of the diet but did not affect digestive enzyme activity or apparent digestibility of amino acids

In the early life stage of broilers, the digestive system is not fully developed, as it cannot secrete enough gastric acid and digestive enzymes. This results in a high pH value in the gastrointestinal tract and proliferation of harmful microorganisms [33]. Therefore, it should be ensured that the starter diet of broilers has a low acid binding capacity to reduce the pH value of the intestine. Our results showed that the pH and ABC values of the broiler starter diet can be decreased by adding SPC to the starter diet. This finding indicates that supplementing a starter diet with SPC is beneficial to maintaining the intestinal health of broilers, and our experimental results verify this. The reason being that SPC addition reduces the pH value during processing allowing diet adaptation to the early growth and development of broilers. Consistent with the results of this study, previous studies also showed that the addition of SPC to the starter diet could reduce the ABC value [20]. In this study, SPC supplementation in the starter diet did not change the pancreatic digestive enzyme activity of broilers. In contrast to the results of this study, Feng et al. [34] and Sun et al. [35] showed that adding modified soybean meal to broiler diets increased the activities of digestive enzymes (trypsin, lipase and protein) in broilers. Trypsin and chymotrypsin activity depended on the protein source [36], but in the current study, the dietary protein source was the same in all treatments.

The results obtained using the faecal collection method to calculate the digestibility of amino acids do not accurately reflect the actual digestibility of amino acids because caecal microbes can utilize undigested dietary protein [37]. Measuring the digestibility of amino acids in the terminal ileum will more truly reflect the ability of the animal to digest and absorb amino acids, as most amino acids are digested and absorbed in the small intestine [38]. The results of this study showed that the addition of 12% SPC to the starter diet did not change the apparent digestibility of ileal terminal amino acids, and the results of Park et al. [39] also showed that 43% and 47% soybean meal addition did not change the apparent digestibility of ileal terminal amino acids in broilers. Navarro et al. [40] compared the apparent digestibility of amino acids of hydrolysed soybean protein, expanded soybean meal, SPC and dehulled soybean meal at the terminal ileum of pigs. The results showed that enzymatically hydrolysed soybean protein, expanded soybean meal and SPC did not change the apparent digestibility of amino acids, while dehulled soybean meal increased the digestibility of proline, and there was no difference in the apparent digestibility of other amino acids. The digestibility of amino acids is affected by many factors, such as digestibility, animal age, feed type and content of digestible amino acids in feed ingredients [41]. This partly explains the improvement of production performance in this experiment, but the apparent digestibility of amino acids was not improved; that is, the improvement of production performance of broilers by SPC may not be achieved by improving the apparent digestibility of amino acids in the terminal ileum.

### Supplementing SPC in the starter diet of broilers can promote intestinal development

The small intestine is the largest digesting and absorbing organ of the body and plays a major role in the process of nutrient digestion and assimilation [42]. Early intestinal development is crucial for chickens to maximize their growth potential [43]. Therefore, any improvement in early intestinal morphology and function is conducive to the improvement of broiler production performance [43, 44]. The morphological characteristics of the small intestine, including villus height, villus width, villus surface area and crypt depth, are all functional indicators of the small intestine [45, 46]. The increase in villus height indicates better growth of broilers because an increase in villus height indicates an increase in the number of intestinal epithelial cells, which is beneficial to the growth of broilers [47, 48]. The results showed that the starter diet supplemented with 12% SPC increased the jejunum villus height and ileum muscle thickness of broilers at d 10. The research of Jazi et al. [49] showed that exchanging soybean meal with fermented cottonseed meal increased the villus height of the jejunum in broilers, which is consistent with the results of this experiment. In addition, Nabizadeh et al. [50] reported that soy protein isolate supplementation in the diet could increase the villus height of the duodenum, jejunum and ileum of broilers at d 10. This may be due to the significant decrease in the level of antinutrient factors and the content of no starch polysaccharides in SPC and soy protein isolate after processing.

The neutral and acidic mucins secreted by goblet cells are important components of the intestinal mucus layer [51]. As part of the inherent host response system, the mucus layer protects epithelial cells from harmful components in the intestinal cavity. It plays a role in nutritional absorption and prevention of intestinal diseases [52]. Our results showed that 12% SPC supplementation in the starter diet increased the number of goblet cells in the duodenum at d 10. Wang et al. [53] also showed that dietary supplementation with N-acetyl-L-glutamate increased the density of duodenal goblet cells in broilers at d 7. In addition, studies have also reported that a diet rich in fibre can increase the number of goblet cells in the chicken intestine [54, 55]. Together with our results, these results show that it is possible through the diet to enhance the immune ability of the broilers.

### Adding SPC to the starter diet can improve the performance of broilers by changing the composition and abundance of intestinal microorganisms and increasing the content of beneficial metabolites

The caecum is the main area of intestinal microbial activity in poultry, and is also the main organ used to measure intestinal microbial abundance in broilers [56]. The balance between beneficial bacteria and pathogenic bacteria in the intestine can be achieved by stimulating the growth of beneficial bacteria and reducing the growth of pathogenic bacteria. This balance between microorganisms can improve the feed efficiency of broilers and improve their production performance [57]. The higher the abundance of caecal microflora, the better the growth performance of broilers [58]. Our results showed that SPC increased the Chao1 and ACE indices of broilers at d 10 and changed the composition of intestinal microorganisms, which indicated that the abundance of gut microbes in the early growth phase increased to some extent [59]. In accordance with the results of this study, Xie et al. [60] also showed that the addition of fermented soybean meal to the diet increased the abundance of intestinal microorganisms.

Consumption of soybeans or soy foods may change the composition and abundance of intestinal microflora [17, 61]. It has been reported that soybean protein can be used as a nitrogen and carbon source for microorganisms to support bacterial growth in the intestinal tract and maintain their life activities [62]. In this study, the microorganisms of TOP30 in 10-day-old broilers were analysed. The results showed that 12% SPC supplementation could increase the relative abundance of *Alistipes*, *Anaerotruncus*, *Erysipelatoclostridium*, *Intestinimonas*, *Flavonifractor* and *Marvinbryantia*. Previous studies have shown that *Alistipes* [63], *Anaerotruncus* [64], *Erysipelatoclostridium* [65], *Intestinimonas* [66], *Flavonifractor* [67, 68] and *Marvinbryantia* [69] are SCFA-producing microorganisms. Among those, *Alistipes* [70], *Anaerotruncus* [71], *Erysipelatoclostridium* [65], *Intestinimonas* [66] and *Flavonifractor* [67] were all related to the increase in butyric acid content. *Erysipelatoclostridium* increased butyrate content by fermenting complex dietary carbohydrates [65]. *Flavonifractor* was first isolated from the human intestine and can convert quercetin or other flavonoids into acetic acid and butyric acid [67]. *Intestinimonas* can produce butyric acid from sugars and amino acids, which can effectively convert lysine to butyrate and acetic acid [66]. This characteristic is unique to the intestinal ecosystem. It connects two important metabolic characteristics, namely, butyric acid production and amino acid fermentation in the intestinal tract.

The function of our differentially abundant bacteria shows that their abundance is related to changes in the SCFA content. In particular, these five kinds of bacteria can produce butyric acid. SCFAs (acetic acid, propionic acid and butyric acid) are the final products of indigestible dietary components fermented by intestinal microorganisms, which can stimulate epithelial cell regeneration [72]. Acetic acid and propionic acid can inhibit the decomposition of intracellular fat, and butyric acid can improve the barrier function and permeability of intestinal epithelial cells by regulating the expression of tight junction proteins and mucin [73]. The fermentation of fibre in the intestine is related to the increase in butyric acid, and butyric acid may be used as an antibacterial agent against pathogenic bacteria [74]. Therefore, the production of butyric acid may promote intestinal health. For this reason, we determined the SCFAs content in the caecum, and the results were also interesting. The addition of SPC to the starter diet increased the content of butyric acid and total SCFAs in the caecum of broilers, which was consistent with the increase in the relative abundance of the intestinal flora. Therefore, we speculate that the addition of SPC to the starter diet increased the content of butyric acid by stimulating the relative abundance of *Alistipes*, *Anaerotruncus*, *Erysipelatoclostridium*, *Intestinimonas* and *Flavonifractor*, thus improving the production performance of broilers and promoting their intestinal development to some extent. The results of the correlation analysis support further this argument. The results showed that the increase in the abundance of *Flavonifractor* was consistent with the increase in butyric acid content and the decrease in jejunal length and relative weight. In addition, the increase in the abundance of *Intestinimonas* was consistent with the increase in the content of total short-chain fatty acids sand the decrease in the relative weight of the jejunum. Therefore, we speculate that the increase in the abundance of *Alistipes, Anaerotruncus, Erysipelatoclostridium, Intestinimonas* and *Flavonifractor* is very important for the starting growth of broilers.

## Conclusion

In conclusion, dietary SPC supplementation in the early diet can promote the early growth of broilers to promote their total life growth. This growth-promoting effect is achieved by promoting intestinal development and increasing the content of short-chain fatty acids by promoting the abundance of intestinal probiotics. Therefore, SPC can be used as an accelerator for early growth.

## Supplementary Information

**Additional file 1: Table S1.** Amino acid and protein levels in the diet (dry matter basis). **Table S2.** Effects of dietary SPC in the starter diet on the intestinal pH value of broilers at 10 d. **Table S3**. Effects of dietary SPC in the starter diet on the relative abundance of species at the TOP30 genus level of caecal microbes in broilers at 10 d. **Table S4.** Effects of dietary SPC in the starter diet on the relative abundance of species at the TOP30 genus level of caecal microbes in broilers at 42 d. **Fig. S1. **Effects of SPC in the starter diet on the growth performance of broilers. The values in the histogram are the means ± SEM (*n* = 8), *P < 0.05. Control = control group; SPC12 = supplement 12% SPC to the starter diet group; F/G = average daily feed intake/average daily gain.

## Data Availability

The datasets used and/or analysed during the current study are available from the corresponding author upon reasonable request. The 16S rRNA sequencing data of the manuscript have been uploaded to the NCBI database. The login number of the 10-day-old sequencing data is PRJNA783362, and the login number of the 42-day-old sequencing data is PRJNA783369.

## Abbreviations

SPC: Soy protein concentrate
ABC: Acid-binding capacity
GP: General proteolytic
VH: Villus height
CD: Crypt depth
VCR: Villus height to crypt depth ratio
OTU: Operational taxonomic units
PCR: Polymerase chain reaction
PCoA: Principal coordinates analysis
LEfSe: The linear discriminant analysis effect size analysis
SCFA: Short-chain fatty acid
SPSS: Statistical package for social science
