# Supplementary material for: Supplementing the early diet of broilers with soy protein concentrate can improve intestinal development and enhance short-chain fatty acid-producing microbes and short-chain fatty acids, especially butyric acid
Source: J Anim Sci Biotechnol. 2022 Sep 8;13:97. doi: 10.1186/s40104-022-00749-5 (PMC9454139; doi:10.1186/s40104-022-00749-5)
Supplement: Supplementary file 1 — Additional file 1: Table S1. Amino acid and protein levels in the diet (dry matter basis). Table S2. Effects of dietary SPC in the starter diet on the intestinal pH value of broilers at 10 d. Table S3. Effects of dietary SPC in the starter diet on the relative abundance of species at the TOP30 genus level of caecal microbes in broilers at 10 d. Table S4. Effects of dietary SPC in the starter diet on the relative abundance of species at the TOP30 genus level of caecal microbes in broilers at 42 d. Fig. S1. Effects of SPC in the starter diet on the growth performance of broilers. The values in the histogram are the means ± SEM (n = 8), *P < 0.05. Control = control group; SPC12 = supplement 12% SPC to the starter diet group; F/G = average daily feed intake/average daily gain. [file 40104_2022_749_MOESM1_ESM.zip › Supplement material.docx]

**Supplement Tables**

Table S1 Amino acid and protein levels in diet (dry matter basis)

| Items | Phases | | | |
| --- | --- | --- | --- | --- |
|  | 1-10 d | | 11-22 d | 22-42 d |
|  | control | SPC12 |  |  |
| Dry matter, % | 86.62 | 86.19 | 88.69 | 90.21 |
| Crude protein, % | 26.18 | 26.19 | 23.17 | 21.17 |
| Methionine, % | 0.65 | 0.65 | 0.70 | 0.60 |
| Cystine, % | 0.41 | 0.45 | 0.37 | 0.34 |
| Methionine + Cystine, % | 1.06 | 1.10 | 1.07 | 0.95 |
| Lysine, % | 1.54 | 1.53 | 1.45 | 1.22 |
| Threonine, % | 1.00 | 1.03 | 0.86 | 0.82 |
| Arginine, % | 1.80 | 1.86 | 1.50 | 1.41 |
| Isoleucine, % | 1.14 | 1.18 | 0.89 | 0.89 |
| Leucine, % | 2.20 | 2.35 | 1.85 | 1.82 |
| Valine, % | 1.28 | 1.32 | 1.02 | 0.99 |
| Histidine, % | 0.72 | 0.74 | 0.62 | 0.58 |
| Phenylalanine, % | 1.33 | 1.40 | 1.09 | 1.08 |
| Tyrosine, % | 0.90 | 0.99 | 0.83 | 0.77 |
| Glycine, % | 1.14 | 1.15 | 0.93 | 0.88 |
| Serine, % | 1.27 | 1.35 | 1.10 | 1.05 |
| Proline, % | 1.41 | 1.49 | 1.25 | 1.14 |
| Alanine, % | 1.29 | 1.34 | 1.09 | 1.07 |
| Aspartate, % | 2.78 | 2.86 | 2.27 | 2.15 |
| Glutamate, % | 4.59 | 4.76 | 3.82 | 3.71 |
| Total amino acids ammonia, % | 25.00 | 25.90 | 21.12 | 20.19 |
| Total amino acids, % | 24.66 | 25.55 | 20.86 | 19.81 |

Table S2 Effects of dietary SPC in starter diet on intestine pH value of broilers at 10 d

| Items | Group | | *P-*value |
| --- | --- | --- | --- |
|  | Control | SPC12 |  |
| Duodenum pH | 6.26±0.36 | 6.00±0.36 | 0.388 |
| Jejunum pH | 5.82±0.05 | 5.99±0.12 | 0.080 |
| Ileal pH | 5.42±0.13 | 5.47±0.17 | 0.979 |
| Crop pH | 4.24±0.14 | 4.41±0.29 | 0.392 |
| Gizzard pH | 3.34±0.16 | 3.42±0.26 | 0.106 |
| Glandular stomach pH | 2.80±0.16 | 2.66±0.19 | 0.269 |

Table S3 Effects of dietary SPC in starter diet on relative abundance of species at the TOP30 Genus level of cecal microbe in broilers at 10 d

| Taxonomy | | | Groups | | *P*-value |
| --- | --- | --- | --- | --- | --- |
| Phyla classification | Family classification | Genus classification | Control | SPC12 |  |
| Firmicutes | Ruminococcaceae | *Faecalibacterium* | 0.26465±0.056914 | 0.18038±0.038003 | 0.238 |
|  |  | *unidentified_Ruminococcaceae* | 0.11744±0.018107 | 0.07830±0.010297 | 0.081 |
|  |  | *Butyricicoccus* | 0.01061±0.002428 | 0.01180±0.002000 | 0.709 |
|  |  | *Fournierella* | 0.00076±0.000322 | 0.00101±0.000412 | 0.633 |
|  |  | *Subdoligranulum* | 0.00273±0.001012 | 0.00915±0.002824 | 0.062 |
|  |  | *Anaerotruncus* | 0.00316±0.000787 | 0.00957±0.002404 | 0.033 |
|  |  | *Intestinimonas* | 0.00226±0.000201 | 0.00430±0.000409 | 0.001 |
|  |  | *Negativibacillus* | 0.00210±0.000087 | 0.00299±0.000442 | 0.087 |
|  |  | *Oscillibacter* | 0.00105±0.000193 | 0.00148±0.000074 | 0.069 |
|  |  | *Flavonifractor* | 0.00060±0.000060 | 0.00086±0.000074 | 0.018 |
|  | Lachnospiraceae | *unidentified_Lachnospiraceae* | 0.05498±0.005867 | 0.09061±0.016029 | 0.067 |
|  |  | *Sellimonas* | 0.00586±0.000713 | 0.00849±0.001070 | 0.061 |
|  |  | *Shuttleworthia* | 0.00461±0.000486 | 0.00898±0.002364 | 0.110 |
|  |  | *Lachnoclostridium* | 0.00545±0.000811 | 0.00861±0.001653 | 0.116 |
|  |  | *Blautia* | 0.00488±0.000886 | 0.00768±0.001281 | 0.094 |
|  |  | *Tyzzerella* | 0.00611±0.001295 | 0.00692±0.000989 | 0.629 |
|  |  | *Eisenbergiella* | 0.00309±0.000526 | 0.00443±0.000581 | 0.109 |
|  |  | *Anaerostipes* | 0.00148±0.000456 | 0.00099±0.000126 | 0.336 |
|  |  | *Marvinbryantia* | 0.00046±0.000076 | 0.00104±0.000038 | 0.000 |
|  | Lactobacillaceae | *Lactobacillus* | 0.07367±0.013427 | 0.04028±0.007608 | 0.058 |
|  | unidentified_Clostridiales | *unidentified_Clostridiales* | 0.00709±0.003564 | 0.00337±0.001183 | 0.349 |
|  | Erysipelotrichaceae | *Erysipelatoclostridium* | 0.00262±0.000423 | 0.00518±0.000975 | 0.025 |
|  | Enterococcaceae | *Catellicoccus* | 0.00001±0.000004 | 0.00001±0.000010 | 0.764 |
|  |  | *Enterococcus* | 0.00133±0.000428 | 0.00126±0.000322 | 0.894 |
|  | unidentified_Clostridiales | *Candidatus_Arthromitus* | 0.00021±0.000035 | 0.00025±0.000050 | 0.519 |
| Bacteroidetes | Bacteroidaceae | *Bacteroides* | 0.01650±0.004965 | 0.00844±0.002519 | 0.177 |
|  | Rikenellaceae | *Alistipes* | 0.02261±0.005890 | 0.05230±0.006925 | 0.007 |
| Tenericutes | Anaeroplasmataceae | *Anaeroplasma* | 0.00157±0.000695 | 0.00340±0.001008 | 0.170 |
| Proteobacteria | Enterobacteriaceae | *unidentified_Enterobacteriaceae* | 0.00305±0.000574 | 0.00579±0.001411 | 0.112 |
|  |  | *Plesiomonas* | 0.00034±0.000259 | 0.00004±0.000032 | 0.296 |

Table S4 Effects of dietary SPC in starter diet on relative abundance of species at the TOP30 Genus level of cecal microbe in broilers at 42 d

| Taxonomy | | | Groups | | *P*-value |
| --- | --- | --- | --- | --- | --- |
| Phyla classification | Family classification | Genus classification | Control | SPC12 |  |
| Firmicutes | Ruminococcaceae | *Faecalibacterium* | 0.05698±0.008952 | 0.06604±0.012912 | 0.574 |
|  |  | *unidentified_Ruminococcaceae* | 0.03661±0.003416 | 0.04568±0.001683 | 0.032 |
|  |  | *Butyricicoccus* | 0.01871±0.003625 | 0.01677±0.003037 | 0.694 |
|  |  | *Negativibacillus* | 0.00856±0.001276 | 0.00881±0.000472 | 0.862 |
|  |  | *Oscillibacter* | 0.00661±0.000543 | 0.00755±0.000392 | 0.186 |
|  |  | *Flavonifractor* | 0.00623±0.001196 | 0.00617±0.000355 | 0.959 |
|  |  | *Intestinimonas* | 0.00548±0.000791 | 0.00703±0.000771 | 0.182 |
|  | Lachnospiraceae | *unidentified_Lachnospiraceae* | 0.05011±0.010586 | 0.04344±0.003353 | 0.558 |
|  |  | *Eisenbergiella* | 0.02354±0.002077 | 0.02322±0.002777 | 0.927 |
|  |  | *Shuttleworthia* | 0.00987±0.002041 | 0.01254±0.002260 | 0.402 |
|  |  | *Lachnoclostridium* | 0.01253±0.001918 | 0.01080±0.001054 | 0.441 |
|  |  | *Tyzzerella* | 0.00637±0.000545 | 0.00790±0.001306 | 0.309 |
|  |  | *Sellimonas* | 0.00275±0.000559 | 0.00356±0.000775 | 0.408 |
|  |  | *Blautia* | 0.00441±0.000638 | 0.00396±0.000494 | 0.587 |
|  |  | *Lactobacillus* | 0.01181±0.002417 | 0.00896±0.003007 | 0.475 |
|  | Christensenellaceae | *unidentified_Christensenellaceae* | 0.00374±0.000472 | 0.00385±0.000652 | 0.894 |
|  | Acidaminococcaceae | *Phascolarctobacterium* | 0.00545±0.001712 | 0.00291±0.001223 | 0.261 |
|  | unidentified_Clostridiales | *unidentified_Clostridiales* | 0.00863±0.001050 | 0.00929±0.001184 | 0.681 |
| Bacteroidetes | Bacteroidaceae | *Bacteroides* | 0.01401±0.003829 | 0.02101±0.005934 | 0.355 |
|  |  | *Barnesiella* | 0.08387±0.040549 | 0.06303±0.035330 | 0.704 |
|  | Marinifilaceae | *Odoribacter* | 0.00318±0.000694 | 0.00467±0.001603 | 0.415 |
|  | Rikenellaceae | *Alistipes* | 0.11680±0.026416 | 0.09419±0.014185 | 0.463 |
|  |  | *Rikenella* | 0.00249±0.000653 | 0.00654±0.001992 | 0.087 |
|  | Tannerellaceae | *Parabacteroides* | 0.00591±0.001584 | 0.00306±0.000745 | 0.127 |
| Proteobacteria | Pseudomonadaceae | *Pseudomonas* | 0.00715±0.002135 | 0.01096±0.000760 | 0.137 |
|  | Burkholderiaceae | *Parasutterella* | 0.00427±0.000520 | 0.00525±0.000974 | 0.413 |
|  | Desulfovibrionaceae | *Bilophila* | 0.00440±0.001326 | 0.00275±0.000308 | 0.260 |
|  | Enterobacteriaceae | *unidentified_Enterobacteriaceae* | 0.00173±0.000563 | 0.00105±0.000208 | 0.278 |
| unidentified_Bacteria | Helicobacteraceae | *Helicobacter* | 0.00308±0.000887 | 0.00806±0.002263 | 0.076 |
|  | Campylobacteraceae | *Campylobacter* | 0.00026±0.000092 | 0.00014±0.000029 | 0.269 |
